# Verification of prognostic expression biomarkers is improved by examining enriched leukemic blasts rather than mononuclear cells from acute myeloid leukemia patients

**DOI:** 10.1186/s40364-023-00461-0

**Published:** 2023-03-16

**Authors:** Era L. Pogosova-Agadjanyan, Xing Hua, Megan Othus, Frederick R. Appelbaum, Thomas R. Chauncey, Harry P. Erba, Matthew P. Fitzgibbon, Isaac C. Jenkins, Min Fang, Stanley C. Lee, Anna Moseley, Jasmine Naru, Jerald P. Radich, Jenny L. Smith, Brooke E. Willborg, Cheryl L. Willman, Feinan Wu, Soheil Meshinchi, Derek L. Stirewalt

**Affiliations:** 1 Clinical Research Division, Fred Hutchinson Cancer Center, 1100 Fairview Ave N, D5-112, Seattle, WA 98109 USA; 2 SWOG Statistical Center, Fred Hutchinson Cancer Center, Seattle, WA USA; 3 Departments of Oncology and Hematology, University of Washington, Seattle, WA USA; 4 VA Puget Sound Health Care System, Seattle, WA USA; 5 Duke Cancer Institute, Durham, NC USA; 6 Bioinformatics Shared Resource, Fred Hutchinson Cancer Center, Seattle, WA USA; 7 Clinical Biostatistics, Fred Hutchinson Cancer Center, Seattle, WA USA; 8 Department of Pediatrics, University of Washington, Seattle, WA USA; 9 Department of Laboratory Medicine and Pathology, Mayo Clinic Comprehensive Cancer Center, Rochester, MN USA

**Keywords:** Biomarkers, Prognostic biomarkers, Genetic biomarkers, Hematological cancers, Leukemias, Acute myeloid leukemia, Transcriptome, Translational research

## Abstract

**Background:**

Studies have not systematically compared the ability to verify performance of prognostic transcripts in paired bulk mononuclear cells versus viable CD34-expressing leukemic blasts from patients with acute myeloid leukemia. We hypothesized that examining the homogenous leukemic blasts will yield different biological information and may improve prognostic performance of expression biomarkers.

**Methods:**

To assess the impact of cellular heterogeneity on expression biomarkers in acute myeloid leukemia, we systematically examined paired mononuclear cells and viable CD34-expressing leukemic blasts from SWOG diagnostic specimens. After enrichment, patients were assigned into discovery and validation cohorts based on availability of extracted RNA. Analyses of RNA sequencing data examined how enrichment impacted differentially expressed genes associated with pre-analytic variables, patient characteristics, and clinical outcomes.

**Results:**

Blast enrichment yielded significantly different expression profiles and biological pathways associated with clinical characteristics (e.g., cytogenetics). Although numerous differentially expressed genes were associated with clinical outcomes, most lost their prognostic significance in the mononuclear cells and blasts after adjusting for age and ELN risk, with only 11 genes remaining significant for overall survival in both cell populations (*CEP70*, *COMMD7*, *DNMT3B*, *ECE1*, *LNX2*, *NEGR1*, *PIK3C2B*, *SEMA4D*, *SMAD2*, *TAF8*, *ZNF444*). To examine the impact of enrichment on biomarker verification, these 11 candidate biomarkers were examined by quantitative RT/PCR in the validation cohort. After adjusting for ELN risk and age, expression of 4 genes (*CEP70*, *DNMT3B*, *ECE1*, and *PIK3CB*) remained significantly associated with overall survival in the blasts, while none met statistical significance in mononuclear cells.

**Conclusions:**

This study provides insights into biological information gained/lost by examining viable CD34-expressing leukemic blasts versus mononuclear cells from the same patient and shows an improved verification rate for expression biomarkers in blasts.

**Supplementary Information:**

The online version contains supplementary material available at 10.1186/s40364-023-00461-0.

## Background

AML is one of the most common and deadly hematopoietic malignancies. Like many cancers, the incidence of AML increases with age, such that the median age at diagnosis is 68 years. Patients with AML can receive a variety of different therapies, ranging from disease modifying agents to myeloablative allogeneic transplants. When deciding optimal care, physicians and patients must consider multiple factors, including age, performance status, and likelihood of a favorable response to therapy. Over the last three decades, many prognostic biomarkers have been identified for adult patients with AML. These prognostic biomarkers have been incorporated into the European LeukemiaNet (ELN) risk classification [1], which remains the gold standard for prognostication of patients with AML [2, 3].

ELN risk is currently determined by a combination of cytogenetics, selective mutations, and preceding predisposition to the development of AML (i.e., history of myelodysplasia, etc.). Gene expression forms one of the major cornerstones supporting the intrinsic biology of leukemic cells, with transcription being regulated by multiple genetic and epigenetic regulators. Although multiple expression biomarkers and profiles have been shown to correlate with clinical outcome, none are currently utilized in ELN risk stratification for AML [4–18]. There are likely multiple reasons for the lack of expression biomarkers translating into clinical practice, ranging from problems with reproducibility to technical issues for implementing assays in clinical setting. Nevertheless, given the importance of transcription, it seems that transcript biomarkers would hold the potential promise to inform and improve upon ELN risk classification in the clinical setting – especially if we could identify means to make these transcript biomarkers more reliable and reproducible.

**Fig. 1:**
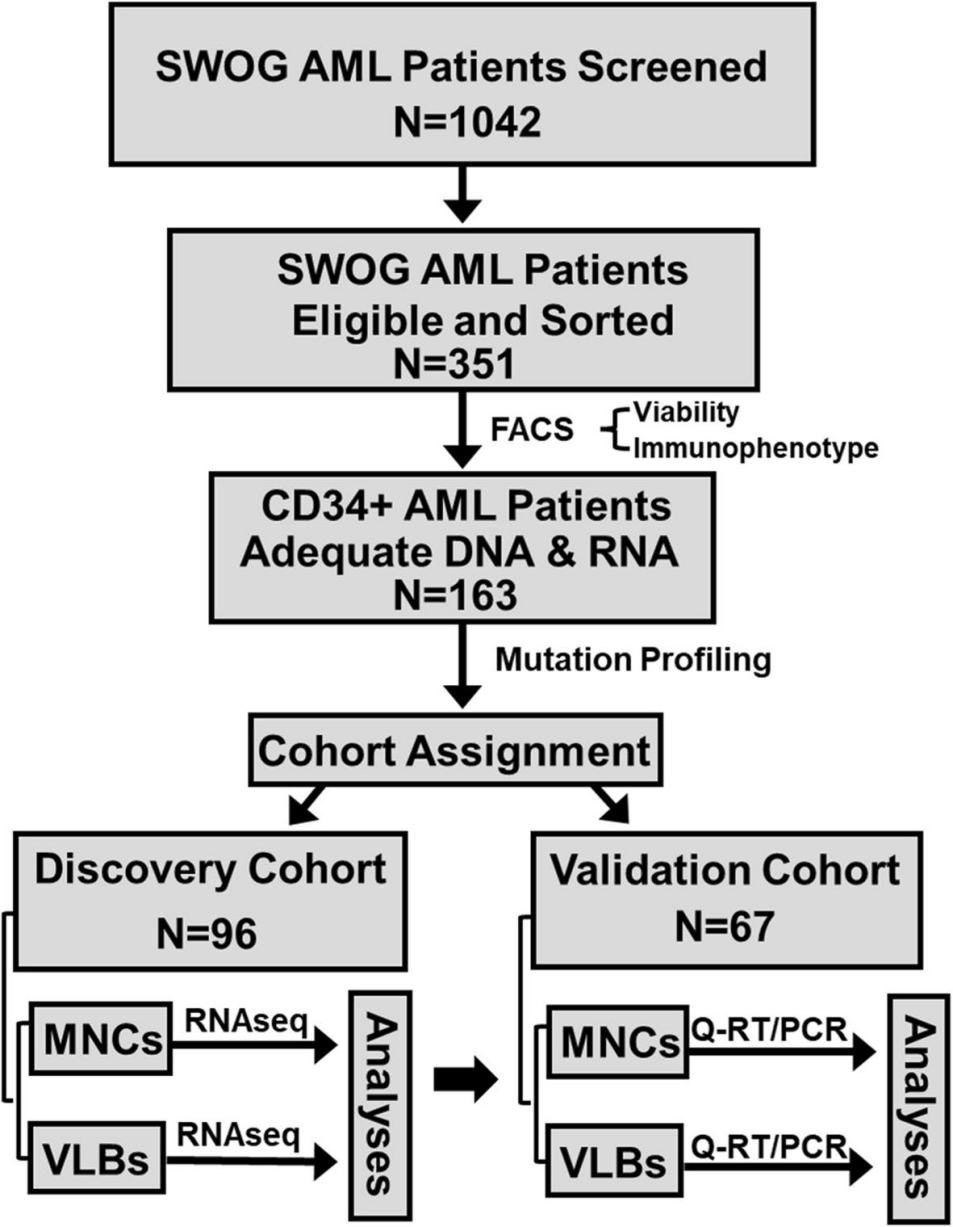
Overview of the study. Figure provides the workflow for the overall study

Current biomarker assays primarily examine either total nucleated cells or bulk mononuclear cells (MNCs), with the leukemic blast percentage varying from 20 to 100% in AML. This inter-specimen variability alters the quantitative expression [19], which likely impacts results of analyses investigating differentially expressed genes (DEGs). We hypothesized that eliminating the dying, non-leukemic and differentiated AML blasts (CD34-) may provide a unique window into the biology arising from known clinical prognostic factors, while potentially improving the prognostic performance of expression biomarkers. Therefore, we systematically examined the transcriptomes of paired bulk MNCs and viable leukemic blasts expressing CD34 (VLBs^CD34+^) from diagnostic AML specimens (Fig. 1). We focused on patients with CD34+ leukemia (AML^CD34+^), the most common immunophenotype in AML, to facilitate the enrichment of less differentiated VLBs (i.e., CD34+) and improve homogeneity of the examined cells. Furthermore, we included patients across a broad range of ages to examine the potential impact of age on the results, given that most current biomarker studies have been limited to younger patients and many of these prognostic biomarkers are less informative for older patients [3, 20, 21]. Analyses identified DEGs associated with sample source (blood vs. marrow), cell populations (MNCs vs. VLBs^CD34+^), clinical characteristics, and outcomes. Pathway analyses showed that the information derived from the transcriptome was dependent on the studied cell population. Adjusting for age and ELN risk eliminated most DEGs associated with prognosis in univariate analyses, allowing to focus verification efforts on a select number of genes. Studies examining these prognostic DEGs in an independent cohort of patients showed a higher rate of verification using RNA from the VLBs^CD34+^ than bulk MNCs.

## Methods

### Patient materials

A review of SWOG Cancer Research Network leukemia repository inventory identified 351 out of 1042 previously untreated AML patients with pretreatment samples potentially containing enough cryopreserved vials for the proposed studies and who received intensive therapy with curative intent. Patients were enrolled onto protocols SWOG-9031, SWOG-9333, S0106 and S0112 and treated as previously described [22–25]. Specimen handling and cryopreservation were consistent across the trials as previously described [3]. All participants provided written informed consent in compliance with the Declaration of Helsinki, and studies were conducted with the approval of the Fred Hutchinson Cancer Center Institution Review Board.

### Thawing, fluorescence-activated cell sorting (FACS), and nucleic acid extraction

Cryopreserved samples were thawed as previously described [3, 19]. A portion of bulk MNCs was lysed, while the remainder underwent FACS to isolate VLBs^CD34+^ using forward by side scatter, DAPI staining and fluorescently-labeled antibodies to CD45, CD34, CD38, and CD117 as previously described [3, 19]. DNA and RNA were extracted from the bulk MNCs and VLBs^CD34+^, quantified and assessed for quality as previously described [3, 19].

### Identification of genomic mutations

Internal tandem duplications in *FLT3* (*FLT3*-ITDs) were identified and censored at an upper limit of 20 as previously described [20, 26]. TruSight™ Myeloid Sequencing Panel (Illumina, San Diego, CA, USA) was used for DNA sequencing. TruSight™ platform provided inadequate coverage for *CEBPA* and *NRAS* Exon 3; therefore, in-house targeted MiSeq assays were developed to cover these loci (Table S1A). See Supplemental Methods for additional details regarding alignment, annotation, and quality controls [27–33]. All mutation data have been provided in Tables S1B and C. Nucleotide changes that were not classified as somatic mutations are provided in Table S1D.

### RNA sequencing for transcript biomarkers

RiboErase (Roche, Wilmington, MA, USA) was utilized to deplete ribosomal RNA as per manufacture recommendations. Transcriptome libraries were generated using KAPA Stranded RNA-Seq Library Preparation Kit (KAPA Biosystems/Roche Sequencing Solutions, Inc., Wilmington, MA, USA) [34], and sequenced in batches using either Illumina HiSeq 2500 (HiSeq) or NovaSeq 6000 (NovaSeq) instruments (Illumina, San Diego, CA, USA). Transcripts were mapped, aligned, and quantified using a standard bioinformatic pipeline of software for RNA sequencing (RNAseq) as described in Supplemental Methods [35–45]. Normalized count per million mapped fragment (CPM) and fragments per kilobase of exon per million mapped fragments (FKPM), as well as filtering parameters are provided in Tables S2A-C.

### Quantitative RT/PCR of transcript biomarkers

TaqMan™ gene expression assays were purchased from ThermoFischer Scientific (Waltham, MA, USA). The list of the genes, targeted loci, and assays are provided in Table S3. Transcript expression was quantified as described in Supplemental Methods and previous reports [3, 6, 45].

### Statistical analyses

From the available cohort of 351 patients we expected to have approximately 200 patients with a CD34+ phenotype. Patients were sorted and RNA was extracted sequentially in random order until 96 RNA samples (the size of one plate and approximately half of the expected 200 patients) from patients with CD34+ phenotype were identified and assigned to discovery cohort. While the DNA and RNA analyses in the discovery cohort were ongoing, the remaining samples were extracted and assayed for quality. All patients with CD34+ phenotype were to be included in the second, validation, cohort. A total of 67 patients with CD34+ phenotype had sufficient material for downstream studies.

Cytogenetic risk was categorized per the ELN guidelines [1]. Complete remission (CR) required the following: > 20% marrow cellularity with maturation of all cell lineages, < 5% blasts, no Auer rods, ANC ≥1500/μL, platelets > 100,000/μL, no peripheral blasts, and no extramedullary disease. The one exception was CR for S0106, which required all the previously mentioned characteristics for CR, but utilized ANC ≥1000/μL rather than ANC ≥1500/μL. Overall survival (OS) was measured from the date of study registration to the date of death by any cause, with patients last known to be alive censored at the date of last contact. Quantitative and categorical factors were compared between groups using Wilcoxon Rank-sum tests and Fisher’s exact tests, respectively. OS was estimated using the Kaplan-Meier method and compared between groups using log-rank tests.

Univariate logistic regression and Cox proportional hazard models were used to evaluate the association between CR and OS, respectively, and the log-2 transformed FPKM values of each gene. Multivariable analyses adjusted for gender, age at study registration, cytogenetic risk, ELN risk, or age and ELN risk. Logistic regression models and the Cox proportional hazard models were also used to evaluate associations between the leukemic stem cell 17-biomarker (LSC17) signature and CR and OS, respectively [14].

Significance of RNA expression change was defined as a combination of FDR and either fold change (FC), odds ratio (OR) or hazard ratio (HR), as appropriate. An FDR < 0.01 and FC > 2 or < 0.5 was considered significant in analyses examining DEGs in paired samples: batch effect, instrument effect, impact of tissue source, and comparisons between MNCs and VLBs^CD34+^. Significant DEGs associated with gender, age, cytogenetic and ELN risk were defined using FDR < 0.1 in combination with following FCs: gender = FC > 2 or < 0.5; age = FC > 1.1 or < 0.9 per unit change (unit for age = 10 years); cytogenetics = FC > 2 or < 0.5 for one or more comparisons between cytogenetic risk groups; and ELN risk = FC > 2 or < 0.5 for one or more comparisons between ELN risk groups. Significant DEGs for clinical outcomes were defined as an FDR < 0.1 and OR > 1.5 or < 0.66 (CR) or HR > 1.5 or < 0.66 (OS). In the validation cohort, significance was defined as *P*-value < 0.05, regardless of clinical effect size.

### Biomolecular pathway analyses

Reactome (http://reactome.org) was utilized as a means to analyze and visualize biomolecular pathways associated with identified sets of genes as previously described [46]. Lists of significant DEGs were uploaded into the Reactome Analysis Tools to analyze the pathways associated with DEGs. Significance for pathway association with gene list was defined as FDR < 0.1 [46].

## Results

### Patient characteristics

Flow cytometry examined diagnostic specimens from 351 AML patients for blast viability and CD34 expression (Fig. 1). Flow studies revealed that samples from 163 patients (46%) expressed CD34 and had adequate amounts of blasts for downstream studies (Table S4A), while samples from 188 patients were excluded due to insufficient VLBs^CD34+^ (*N* = 26) or lack of CD34 expression (*N* = 162). Analyses compared the mutation profiles between included and excluded patients (Fig. S1, Table S4B). The included AML^CD34+^ patients had a lower frequency of *FLT3*-ITD mutations (19 vs. 42%, *P* < 0.001), and fewer mutations in *NPM1* (6 vs. 62%, *P* < 0.001), *DNMT3A* (19 vs. 40%, *P* < 0.001), *RUNX1* (7% vs. 17%, *P* = 0.002), and *TET2* (9 vs. 20%, *P* = 0.002). In addition, the included AML^CD34+^ patients were more likely to be categorized as adverse ELN risk (39 vs. 17%, *P* < 0.001). Despite these differences, the CR and OS were not significantly different between included AML^CD34+^ and excluded patients (Table S4B). These 163 AML^CD34+^ patients with adequate material were assigned into discovery (*N* = 96) and validation (*N* = 67) cohorts (Fig. 1) and similar analyses compared characteristics of patients in two cohorts were done (Table S4C). These comparison analyses showed no significant difference with respect to gender, blast percentages, commonly detected mutations, ELN risk, or CR. OS was slightly higher in the discovery cohort as compared to the validation cohort (*P* = 0.05).

### Impact of batch effect and sequencing instrument on transcriptome

Batch effect can introduce significant expression changes and lead to erroneous results [47]. To assess for the potential impact of batch effect, libraries were prepared at three different time points using RNA from 4 diagnostic MNC specimens and then sequenced using the same instrument. Thirty-nine DEGs showed significant associations with batch effect (Figs.S2A and S3A; Table S5A), all of which represented non-coding RNAs. We also examined the impact of sequencing instruments on DEGs, given the availability and frequent use of HiSeq 2500 vs. NovaSeq 6000 platforms. Libraries were prepared using RNA from MNC specimens (*N* = 5) and sequenced on both instruments. Applying the same definition for significance, 255 DEGs were significantly associated with the sequencing instrument (Figs. S2B and S3B; Table S5B). Again, the vast majority of the DEGs were non-coding (*N* = 246, 96.5%). Overall, 15 DEGs were significant in batch effect and in instrument analyses (overlap).

### Impact of tissue source on transcriptome

Peripheral blood (PB) and bone marrow (BM) specimens are frequently included and analyzed together in AML biomarker studies. Therefore, we investigated the potential impact of tissue source (PB vs. BM) on RNAseq results using paired PB and BM samples from 3 AML patients. For each tissue source, bulk MNCs and VLBs^CD34+^ were examined, providing 4 different RNA sources: MNCs/PB, MNCs/BM, VLBs^CD34+^/PB, and VLBs^CD34+^/BM. Libraries were prepared and sequenced on the HiSeq 2500 instrument. Comparison analyses (PB vs. BM) were performed separately for MNCs and VLBs^CD34+^, identifying 244 significant DEGs in analyses comparing MNCs/PB versus MNCs/BM (Figs. S2C and S3C; Table S5C). The vast majority of the 244 DEGs represented coding genes, either for immunoglobulin-related proteins (*N* = 63, 26%) or other known coding proteins (*N* = 159, 65%). Analyses comparing VLBs^CD34+^/PB versus VLBs^CD34+^/BM identified 53 significant DEGs between the two cell populations, 83% (44/53) representing coding genes (Figs. S2D and S3D; Table S5D). Most of the immunoglobulin-related DEGs from MNC comparisons (MNCs/PB vs. MNCs/BM) were not significant in the VLBs^CD34+^ analyses. Overall, 34 DEGs were significant in both analyses comparing PB versus BM (Table S5E). Most of the overlapping transcripts (29/34, 85%) represented coding genes in a variety of different pathways: apoptosis/cell cycle, cell adhesion, cell signaling, histone maintenance/modification, mitochondria/metabolism, and transcription regulation.

### Expression differences between MNCs and VLBs^CD34+^

**Fig. 2:**
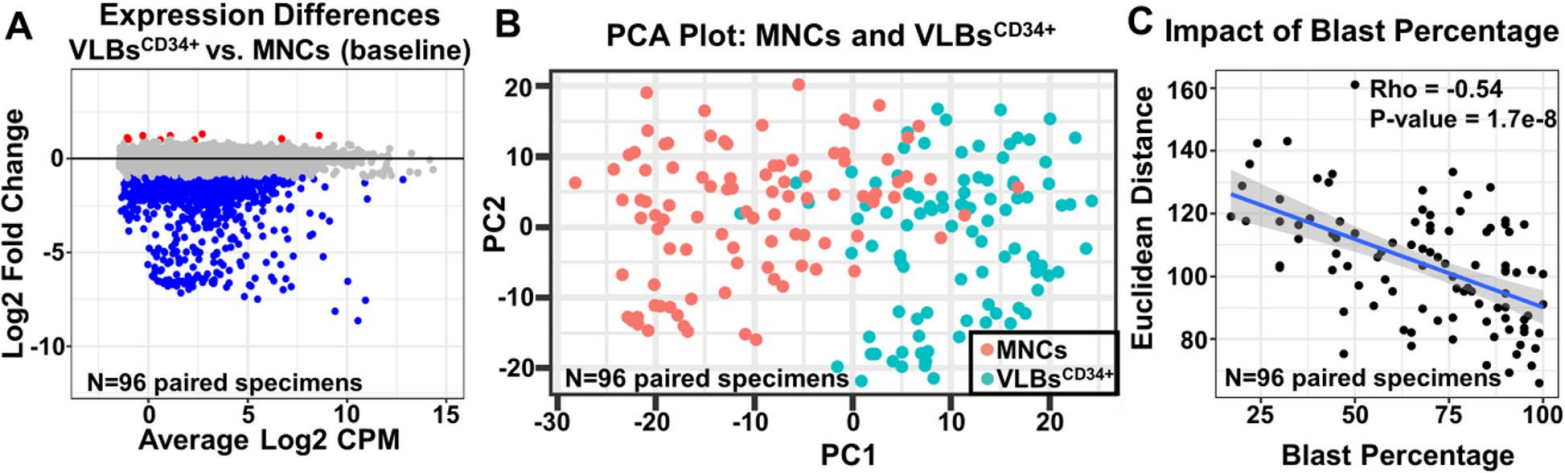
Expression changes between bulk MNCs and VLBs^CD34+^. **A** Figure shows an MD plot displaying log2 fold change in expression profiles between MNCs and VLB^CD34+^. Decreased expression in VLB^CD34+^(blue); Increased expression in VLB^CD34+^ (red). **B** Figure shows a PCA plot showing the variance between paired MNCs and VLB^CD34+^ from 96 specimens. MNCs (light red). VLB^CD34+^ (light blue). **C** Figure shows correlation between Euclidean distance using log2 RPKM values (y-axis) and blast percentage (x-axis). Euclidean distance is used to measure overall transcriptome difference between a pair of MNC and VLB samples

Ninety-six specimens had RNAseq data from paired bulk MNCs and VLBs^CD34+^ (Fig. 1). Transcripts associated with batch effect, sequencing instrument, and tissue source were eliminated from MNCs versus VLBs^CD34+^ analyses, as well as subsequent studies examining associations with clinical characteristics and outcomes. We identified 767 DEGs that were significant between the MNCs and VLBs^CD34+^ (Fig. 2A; Table S5F). These DEGs led to a noticeable shift in the principal component analyses (PCA) plot comparing bulk MNCs and VLBs^CD34+^ (Fig. 2B). Most DEGs between the two cell populations represented coding (*N* = 376, 49%) and immunoglobulin (*N* = 69, 9%) genes. The remainder included pseudogenes (*N* = 180, 23%), snoRNA/snRNA (*N* = 40, 5%), lncRNAs (*N* = 40, 5%), miRNA (*N* = 7, 1%) and other noncoding RNA variants (*N* = 55, 7%). The vast majority of DEGs (758/767, 98.8%) were decreased in the VLBs^CD34+^ relative to the MNCs. Analyses showed a significant negative correlation (Rho = − 0.54, *P*-value < 0.001) between blast percentages and Euclidean distances, indicating that a lower blast percentage resulted in a greater separation between the bulk MNCs and VLBs^CD34+^ (Fig. 2C).

### Transcript correlation with clinical characteristics

Expression data from the paired MNCs and VLBs^CD34+^ (*N* = 96) were examined for DEGs associated with gender, age, WBC, cytogenetic risk, and ELN risk. Gender was significantly associated with expression of a small number of DEGs (MNCs = 12 and VLBs^CD34+^ = 8; overlap = 4; Fig. S4A; Table S6A). Increasing age was significantly associated with 133 and 289 DEGs in the MNCs and VLBs^CD34+^, respectively (overlap = 105, Fig. S4B; Table S6B). There were 392 and 455 DEGs (overlap = 270) significantly associated with cytogenetic risk in MNCs and VLBs^CD34+^, respectively (Fig. S4C; Table S6C), while 168 and 313 DEGs (overlap = 145) were significantly correlated with ELN risk in MNCs and VLBs^CD34+^, respectively (Fig. S4D; Table S6D).

Lists of DEGs significantly associated with age, cytogenetics, and ELN risk were analyzed using Reactome software [46]. Pathways were then ranked from most to least significant by increasing FDR. The lists of age-related DEGs were significantly associated with 2 and 15 pathways using data from the MNCs and VLBs^CD34+^, respectively (Table S7A-C). The top age-related pathway identified in MNCs was ranked 6 in VLBs^CD34+^, while the top age-related pathway in the VLBs^CD34+^ were ranked 237 in the MNCs. For the lists of DEGs significantly associated with cytogenetics, we identified 13 and 4 significant pathways in the MNCs and VLBs^CD34+^, respectively. The top cytogenetic-related pathway in the MNCs was ranked 718 in the VLBs^CD34+^, while the top cytogenetic-related pathways in the VLBs^CD34+^ was ranked 41 in the MNCs (Table S7D-F). Many of the most significant pathways associated with cytogenetics in the MNCs involved lymphocyte function, and their significance, as measured by rank, was substantially diminished by enriching for VLBs^CD34+^, while pathways involving transcription regulation seem to be enriched by examining the VLBs^CD34+^ (Table 1). With respect to the genes associated with ELN risk, the same 2 pathways were significant in both MNCs and VLBs^CD34+^ (Table S7G-I).

**Table 1:** Comparison of significant pathways enriched by DEGs associated with cytogenetic risk in paired bulk MNCs versus VLBs

Cytogenetic-Related Genes in MNCs (*N* = 392)			Cytogenetic-Related Genes in VLBs (*N* = 455)		
Pathway Rank (*N* = 835)	Pathway Name	Entities FDR	Pathway Rank (*N* = 1020)	Pathway Name	Entities FDR
1	Phosphorylation of CD3 and TCR zeta chains	1.09E-11	1	Transcriptional regulation of granulopoiesis	8.70E-05
2	Translocation of ZAP-70 to Immunological synapse	2.46E-11	2	NR1H3 & NR1H2 regulate gene expression linked to cholesterol transport & efflux	0.003861
3	PD-1 signaling	4.86E-11	3	NR1H2 and NR1H3-mediated signaling	0.006610
4	Generation of second messenger molecules	2.46E-09	4	RUNX3 regulates CDKN1A transcription	0.061994
5	Neutrophil degranulation	4.79E-07	13	Neutrophil degranulation	0.450842
6	MHC class II antigen presentation	4.79E-07	53	Cytokine Signaling in Immune system	0.593232
7	Costimulation by the CD28 family	4.94E-07	90	Immune System	0.593232
8	Downstream TCR signaling	6.04E-05	128	Interferon Signaling	0.593232
9	TCR signaling	1.22E-04	359	MHC class II antigen presentation	0.593232
10	Interferon Signaling	7.42E-04	535	Generation of second messenger molecules	0.626337
11	Immune System	7.42E-04	687	Translocation of ZAP-70 to Immunological synapse	0.779539
12	Interferon gamma signaling	0.001212	718	Phosphorylation of CD3 and TCR zeta chains	0.802141
13	Cytokine Signaling in Immune system	0.003510	799	Costimulation by the CD28 family	0.871081
17	RUNX3 regulates CDKN1A transcription	0.107059	828	TCR signaling	0.898506
41	Transcriptional regulation of granulopoiesis	0.500239	991	Interferon gamma signaling	0.998825
55	NR1H2 and NR1H3-mediated signaling	0.557604	Not Identified	PD-1 signaling	N/A
59	NR1H3 & NR1H2 regulate gene expression linked to cholesterol transport & efflux	0.557604	Not Identified	Downstream TCR signaling	N/A

The lists of DEGs significantly associated with cytogenetic risk groups from paired bulk MNCs and VLBs were downloaded into Reactome to identify pathways enriched in the lists. Pathways significantly associated with cytogenetic risk in bulked MNCs and VLBs were examined separately. Table shows the significance and rank by FDR of individual pathways that were significant in MNCs and VLBs using the list derived from bulk MNCs (left columns) and VLBs (right columns). Those pathways significant associated with the list of genes from bulk MNCs and VLBs are highlighted in light blue and gold, respectively. The significant pathways identified in the bulk MNCs (*N* = 13) were not significantly enriched for in the VLBs, and vice versa

### Transcript expression associated with clinical responses

**Fig. 3:**
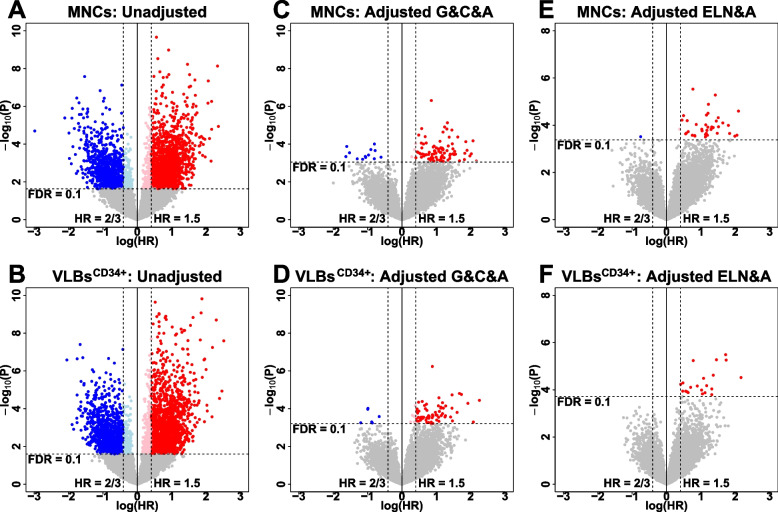
Volcano plots showing transcripts associated with overall survival. Y-axis represents negative Log10 of observed *P*-values. X-axis represents log of the hazard ratio (HR). **A**. Figure shows results for OS in data from MNCs without any adjustment. **B**. Figure shows results for OS in data from VLBs^CD34+^ without any adjustment. **C**. Figure shows results for OS in data from MNCs after adjusting for gender (G), cytogenetic risk (C), and age (A). **D**. Figure shows results for OS in data from VLBs^CD34+^ after adjusting for gender (G), cytogenetic risk (C), and age (A). **E**. Figure shows results for OS in data from MNCs after adjusting for ELN risk (ELN) and age (A). **F**. Figure shows results for OS in data from VLBs^CD34+^ after adjusting for ELN risk (ELN) and age (A). Grey dots represent non-significant transcripts. Red dots represent significant transcripts with an increased expression associated with poor OS. Blue dots represent transcripts with a decreased expression associated with a poor OS.

Univariate analyses identified 3 and 712 DEGs significantly associated with CR in the MNCs and VLBs^CD34+^, respectively (Table S8A). Given the known association of age and cytogenetic risk with CR rates, a multivariable model incorporating age, cytogenetics, and gender was developed to adjust for these variables. After accounting for these variables, none of the transcripts remained significantly associated with CR in either the MNC or VLBs^CD34+^ data (Table S8B). Similarly, all transcripts lost their significance after adjusting for ELN risk and age (Table S8C). Univariate analyses identified total of 2556 and 2678 DEGs significantly associated with OS in the MNCs and VLBs^CD34+^, respectively (overlap = 1771; union = 3463; Fig. 3A and B; Table S8D). Multivariable analyses adjusting for gender, age, and cytogenetic risks identified 101 and 69 transcripts significantly associated with OS in MNCs and VLBs^CD34+^, respectively (overlap = 38; union = 132; Fig. 3C and D; Table S8E). Multivariable analyses adjusting for ELN risk and age identified 38 and 20 transcripts significantly associated with OS in the MNCs and VLBs^CD34+^, respectively (overlap = 14; union = 44; Fig. 3E and F; Table S8F).

### Prognostic significance of the LSC17 in the discovery patients

LSC17 signature has been validated using MNC specimens from pediatric and adult patients with AML [14, 48]. We examined the performance of the LSC17 signature in the paired MNCs and VLBs^CD34+^ using *P*-value < 0.05 to define statistical significance. Univariate analyses showed that the LSC17 model was associated with a reduced CR rate, which was statistically significant in VLBs^CD34+^ data (OR = 0.91, *P*-value = 0.039) and borderline significant in the MNC data (OR = 0.93, *P*-value = 0.119; Table S8). After adjusting for age and ELN risk, the LSC17 signature was no longer significantly associated with CR in either the MNCs or VLBs^CD34+^ (*P*-values = 0.808 and 0.860, respectively; Table S8). The LSC17 signature was significantly associated with OS in MNCs (HR 1.11, *P* = 0.00053) and VLBs^CD34+^ (HR 1.11, *P* = 0.00003), remaining prognostically significant for both the MNCs (HR = 1.07, *P* = 0.023) and VLBs^CD34+^ (HR = 1.07, *P* = 0.00876) after adjusting for age and ELN risk (Fig. S5; Table S9).

### Validation of prognostic transcripts in MNCs and VLBs^CD34+^

**Fig. 4:**
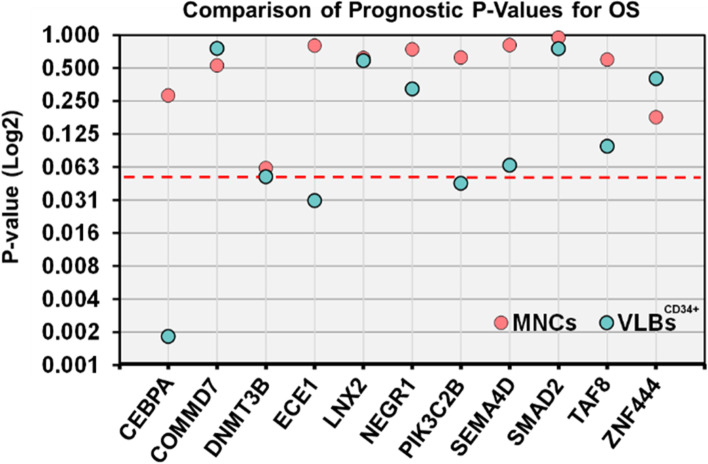
Prognostic *P*-values for overall survival in validation cohort. Y-axis shows the *P*-value for overall survival from the Q-RT/PCR for each gene (x-axis). Light blue represents *P*-value from MNC data. Orange represents *P*-value from VLB^CD34+^ data. The y-axis is log2 transformed to provide a better discrimination for the lower *P*-values. Red Dotted line represents *P*-value=0.05.

For the validation studies, we focused on DEGs that were significant in *both* MNCs and VLBs^CD34+^ after adjusting for ELN risk and age (genes = 14; Table S8G). As a means to validate these DEGs, we chose to employ real-time quantitative reverse transcription/polymerase chain reaction (Q-RT/PCR) assays, which are currently utilized in clinical practice for many diseases, including AML [49]. Therefore, Q-RT/PCR assays were obtained for the coding transcripts (11 of the 14 DEGs), and we examined the association between their RNA expression and OS in paired MNCs and VLBs^CD34+^ from patients in validation cohort (*N* = 67; Fig. 1). After adjusting for age and ELN risk, only *DNMT3B* expression was close to meeting statistical significance for OS in the bulk MNCs (*P* = 0.06, HR = 1.23), while expression for 4 genes (*CEP70*, *DNMT3B*, *ECE1*, and *PIK3CB*) remained significantly associated with OS in the VLBs^CD34+^ (Table 2; Fig. 4). In addition, *SEMA4D* and *TAF8* were borderline significant for adverse OS (*P*-values ≤0.1; Table 2).

**Table 2:** Identification and validation of prognostic transcripts

Gene	MNCs				VLBs			
	RNAseq (*N* = 96)		QRT/PCR (*N* = 67)		RNAseq (*N* = 96)		QRT/PCR (*N* = 67)	
	HR	FDR	HR	*P*-value	HR	FDR	HR	*P*-value
*CEP70*	2.15	0.033	1.14	0.28	2.18	0.018	1.68	< 0.01
*COMMD7*	3.33	0.082	1.15	0.53	4.27	0.018	1.05	0.76
*DNMT3B*	1.57	0.076	1.23	0.06	1.61	0.078	1.30	0.05
*ECE1*	1.78	0.093	1.03	0.80	1.60	0.088	1.32	0.03
*LNX2*	3.38	0.054	1.10	0.62	3.21	0.078	1.08	0.59
*NEGR1*	1.64	0.070	1.02	0.74	1.51	0.078	1.07	0.32
*PIK3C2B*	1.74	0.093	1.06	0.63	1.82	0.088	1.30	0.04
*SEMA4D*	3.13	0.082	0.95	0.81	2.77	0.088	1.38	0.06
*SMAD2*	4.50	0.075	0.99	0.95	5.67	0.018	1.07	0.75
*TAF8*	8.12	0.070	1.12	0.60	8.73	0.058	1.22	0.10
*ZNF444*	4.14	0.033	1.10	0.18	3.06	0.088	1.05	0.40

*MNCs* Bulk mononuclear cells, *VLBs*^*CD34+*^ Undifferentiated CD34+ viable blasts, *RNAseq* RNA sequencing expression results, *Q-RT/PCR* Quantitative RT/PCR assay expression results, *HR* Hazard ratio, *FDR* False discovery rate

## Discussion

The impact of pre-analytic variables and non-leukemic cells on expression remains to be precisely defined but clearly impacts both RNA and protein expression profiling [3, 50]. Our analyses identified a relatively large number of DEGs between paired bulk MNCs and VLBs^CD34+^ (*N* = 767), and as expected, the relative amount of DEGs was inversely correlated with blast percentage (Fig. 2C). By enriching for VLBs^CD34+^, we were able to mitigate the impact of the transcription signal from non-leukemic and dead/dying cells, which also negated some of the impact that tissue source (PB vs. BM) had on the transcriptome. Most of the significant DEGs between MNCs and VLBs^CD34+^ were expressed at lower levels in the VLBs^CD34+^, and not surprisingly, many of the DEGs involved immunoregulatory pathways and/or coded for immunoglobulins. The impact of cellular heterogeneity on the transcription profile was further demonstrated when comparing the biological pathways associated with age and cytogenetic risk. For example, the list of DEGs associated with cytogenetic risk (i.e., cytogenetic-related) primarily enriched for pathways involving lymphocyte signaling in the MNCs, while the cytogenetic-related DEGs in VLBs^CD34+^ enriched for pathways associated with transcription regulation (Table 1). Overall, the results highlight the impact that non-leukemic cells and/or more differentiated leukemic blasts have on transcriptome profile, as well as the information gained and lost by examining bulk MNCs as compared to VLBs^CD34+^. These findings also underscore the importance of examining the appropriate cell populations to answer specific research questions. Given the complexity and differential activity of molecular pathways across hematopoietic cell lineages, studies of mixed populations of cells may impede the ability to dissect out which cell populations are contributing to the biological signal of interest. This phenomenon seemed to be most pronounced when examining potentially biologically meaningful DEGs associated with age and cytogenetics.

Although previous biomarker studies have identified a large number of prognostic transcripts associated with clinical outcomes [4–18], relatively few studies have shown that these transcript profiles remain significant after adjusting for other prognostic factors. Thus, our studies were primarily designed to identify DEGs after adjusting for ELN and age – the two most informative prognostic risk factors for AML [1, 3, 20, 51], while simultaneously examining the potential impact of cellular heterogeneity on DEGs. As with other studies, we identified many prognostically significant DEGs prior to adjusting for age and ELN risk. However, most DEGs lost significance after adjusting for age and ELN risk (Fig. 3). We also examined the prognostic significance of the LSC17 signature, which has been shown to be prognostic for pediatric and adult patients with AML [14, 48]. Unlike most prognostic transcript profiles, the LSC17 was first derived by examining leukemic stem cells and then applied to bulk MNCs. In our analyses, we showed that the LSC17 was statistically associated with OS in both cell sources and remained significant after adjusting for age and ELN risk (Fig. S5). However, the clinical effect size in our analyses was relatively small (HR = 1.07).

Previous prognostic studies have primarily examined the transcriptome in bulk MNCs from patients with AML [4–18, 48], and studies have not systematically compared the ability to verify prognostic DEGs in paired MNCs and VLBs^CD34+^. After adjusting for age and ELN risk, we identified a modest number of coding DEGs (*N* = 11) with comparable statistical significance and clinical effect sizes in both MNCs and VLBs^CD34+^ (Table 2). Focusing on these DEGs, we investigated if examining their expression in VLBs^CD34+^ may improve their verification rate. A targeted approach using real-time Q-RT/PCR was selected for verification since this methodology remains the “gold standard” for independent validation of transcript expression [39, 52] and is currently utilized in clinical practice [49]. In the MNCs, expression of the *DNMT3B* was borderline significant (*P* = 0.061, HR = 1.23), while expressions for the other genes were not statistically significant (Table 2). The results from VLBs^CD34+^ showed a higher rate of prognostic verification. Four DEGs were significantly associated with OS, with all four displaying a HR ≥1.30. Furthermore, the expressions of *SEMA4D* and *TAF8* were borderline significant in VLBs^CD34+^ (Table 2). Some DEGs produced markedly different results using RNA from the paired MNCs and VLBs^CD34+^. For example, *CEP70* was most prognostically significant in VLBs^CD34+^ (*P* = 0.002, HR = 1.68) but failed to meet significance in MNCs (*P* = 0.282, HR = 1.14). Thus, while some DEGs may be informative in MNCs compared to VLBs^CD34+^ or potentially vice versa, our results suggest an increased likelihood of verification and thus, potentially the performance of biomarker assays examining biologically relevant cells. At a minimum, the pathway analyses indicated that biological information elucidated by investigating the transcriptome is different depending upon homogeneity and cell populations.

We focused our analyses on specimens expressing CD34 to facilitate enrichment of a homogenous population of less differentiated VLBs. Given the focus on CD34-expressing leukemia, the results may not be generalizable to other immunophenotypes of AML, and the sample numbers may have limited our ability to identify and verify some prognostic transcripts – especially those with modest clinical effect sizes. Despite these potential limitations, the results show for the first time an increased rate of verification of prognostic biomarkers in enriched leukemic blasts and highlight the challenges of examining heterogenous specimens and the need for additional studies examining the impact of cellular heterogeneity on biomarkers in AML.

## Conclusions

This study provides novel insights into biological information gained/lost by examining bulk MNCs versus VLBs^CD34+^. In addition, the results show a potential benefit for validating expression biomarkers in purified populations of AML blasts. However, additional studies are warranted in larger numbers of samples to verify the relative benefit of biomarker assessment in VLB^CD34+^ and translate findings into clinically compliant assays.

## Supplementary Information

**Additional file 1: Supplemental Methods. Supplemental Figure 1.** Genomic landscape of mutations in various cohorts of patients used for the study. **Supplemental Figure 2.** Quality Control Assessments of RNAseq Data. **Supplemental Figure 3.** Principal Component (PC) Analyses of QC Data. **Supplemental Figure 4.** QQ plots of observed and expected results. **Supplemental Figure 5.** LSC17 score applied to RNAseq data from bulk MNCs and VLBs^CD34+^. **Supplemental Table 1.** Genetic Sequencing Data. **Supplemental Table 2.** RNA Sequencing Data. **Supplemental Table 3.** QRT-PCR Assays. **Supplemental Table 4.** Characteristics of included and excluded patients. **Supplemental Table 5.** Comparisons to assess the impact of batch, instrument, and tissue source on transcript profiles. **Supplemental Table 6.** Expression changes associated with patient characteristics. **Supplemental Table 7.** Pathways associated with DEGs and patient characteristics. **Supplemental Table 8.** Expression changes and pathways associated with clinical outcomes. **Supplemental Table 9.** Prognostic significance of LSC17 in RNA from MNCs and VLBs^CD34+^ from the discovery cohort.

## Data Availability

The datasets generated and/or analyzed during the current study are available in the dbGaP repository, dbGaP under accession number phs002805.v1.p1. Investigators can apply to access sequencing data through standard dbGaP request procedures as described by NIH and found at dbgap_request_process.pdf (nih.gov). Additional data generated or analyzed during this study are included in the supplementary information files.

## Abbreviations

*AML*: Acute Myeloid Leukemia
*AML*^*CD34+*^: Patients with AML whose blasts express CD34+ ell surface antigen
*ANC*: Absolute Neutrophil Count
*BM*: Bone Marrow
*CD*: Cluster of Differentiation
*CD117*: Antibody for proto-oncogene encoding the receptor tyrosine kinase protein known as tyrosine-protein kinase KIT
*CD34*: Antibody for transmembrane phosphoglycoprotein present on hematopoietic stem cells
*CD38*: Antibody for cyclic ADP ribose hydrolase is a glycoprotein found on the surface of many immune cells
*CD45*: Antibody for receptor linked protein tyrosine phosphatase present in all cells of the hematopoietic lineage except erythrocytes and plasma cells
*CEP70*: Centrosomal Protein 70
*CPM*: Count per Million Mapped Fragment
*CR*: Complete Remission
*DAPI*: 4′,6-Diamidino-2-Phenylindole
*DEG*: Differentially Expressed Gene
*DNA*: Deoxyribonucleic Acid
*DNMT3A*: DNA Methyltransferase 3 Alpha
*DNMT3B*: DNA Methyltransferase 3 Beta
*ECE1*: Endothelin Converting Enzyme 1
*ELN*: European LeukemiaNet (ELN) risk classification
*FACS*: Fluorescence-activated Cell Sorting
*FC*: Fold Change
*FDR*: False Discovery Rate
*FKPM*: Fragments per Kilobase of Exon per Million Mapped Fragments
*FLT3*: Fms Related Receptor Tyrosine Kinase 3
*HiSeq*: Illumina HiSeq 2500 Instrument
*HR*: Hazard Ratio
*ITD*: Internal Tandem Duplication
*lncRNAs*: Long Noncoding RNA
*LSC17*: Leukemic Stem Cell 17-biomarker Signature
*miRNA*: MicroRNA
*MNCs*: Mononuclear Cells
*NovaSeq*: Illumina NovaSeq 6000 Instrument
*NPM1*: Nucleophosmin 1
*NRAS*: NRAS Proto-Oncogene, GTPase
*OR*: Odds Ratio
*OS*: Overall Survival
*PB*: Peripheral Blood
*PCA*: Principal Component Analyses
*PIK3CB*: Phosphatidylinositol-4,5-Bisphosphate 3-Kinase Catalytic Subunit Beta
*Q-RT/PCR*: Quantitative Reverse Transcription Polymerase Chain Reaction
*RNA*: Ribonucleic Acid
*RNAseq*: RNA Sequencing
*RT/PCR*: Reverse Transcription Polymerase Chain Reaction
*RUNX1*: RUNX Family Transcription Factor 1
*SEMA4D*: Semaphorin 4D
*snoRNA*: Small Nucleolar RNA
*snRNA*: Small Nuclear RNA
*TAF8*: TATA-Box Binding Protein Associated Factor 8
*TET2*: Tet Methylcytosine Dioxygenase 2
*VLBs*^*CD34+*^: Viable Leukemic Blasts Expressing CD34
*WBC*: White Blood Cell Count
