# Supplementary material for: Verification of prognostic expression biomarkers is improved by examining enriched leukemic blasts rather than mononuclear cells from acute myeloid leukemia patients
Source: Biomark Res. 2023 Mar 16;11:31. doi: 10.1186/s40364-023-00461-0 (PMC10022072; doi:10.1186/s40364-023-00461-0)
Supplement: Supplementary file 1 — Additional file 1: Supplemental Methods. Supplemental Figure 1. Genomic landscape of mutations in various cohorts of patients used for the study. Supplemental Figure 2. Quality Control Assessments of RNAseq Data. Supplemental Figure 3. Principal Component (PC) Analyses of QC Data. Supplemental Figure 4. QQ plots of observed and expected results. Supplemental Figure 5. LSC17 score applied to RNAseq data from bulk MNCs and VLBsCD34+. Supplemental Table 1. Genetic Sequencing Data. Supplemental Table 2. RNA Sequencing Data. Supplemental Table 3. QRT-PCR Assays. Supplemental Table 4. Characteristics of included and excluded patients. Supplemental Table 5. Comparisons to assess the impact of batch, instrument, and tissue source on transcript profiles. Supplemental Table 6. Expression changes associated with patient characteristics. Supplemental Table 7. Pathways associated with DEGs and patient characteristics. Supplemental Table 8. Expression changes and pathways associated with clinical outcomes. Supplemental Table 9. Prognostic significance of LSC17 in RNA from MNCs and VLBsCD34+ from the discovery cohort. [file 40364_2023_461_MOESM1_ESM.zip › Stirewalt_SupplementalMaterials-Updated.docx]

**SUPPLEMENTAL MATERIALS**

**Verification of prognostic expression biomarkers is improved by examining enriched leukemic blasts rather than mononuclear cells from acute myeloid leukemia patients**

**Contents:**

**Supplemental Methods**

Identification of Genomic Mutations

RNA Sequencing for Transcript Biomarkers.

Quantitative RT/PCR of Transcript Biomarkers

**Supplemental Figures**

Supplemental Figure 1: Genomic landscape of mutations in various cohorts of patients used for the study.

Supplemental Figure 2. Quality Control Assessments of RNAseq Data.

Supplemental Figure 3. Principal Component (PC) Analyses of QC Data.

Supplemental Figure 4. QQ plots of observed and expected results.

Supplemental Figure 5. LSC17 score applied to RNAseq data from bulk MNCs and VLBs^CD34+^.

**Supplemental Tables**

Supplemental Table 1: Genetic Sequencing Data (separate file due to size)

Supplemental Table 1A: Targeted Assays for CEBPA and NRAS exon 3

Supplemental Table 1B: All genomic mutations

Supplemental Table 1C: All genomic mutations with ANNOVAR annotations

Supplemental Table 1D: Eliminated SNPs (not classified as somatic mutations)

Supplemental Table 2: RNA Sequencing Data (separate file due to size)

Supplemental Table 2A: Normalized count per million mapped fragment

Supplemental Table 2B: Normalized fragments per kilobase of exon per million mapped fragments

Supplemental Table 2C: Transcript Exclusion

Notes

Supplemental Table 3: QRT-PCR Assays

Supplemental Table 4. Characteristics of included and excluded patients. (separate file due to size)

Supplemental Table 4A. Patient unique identifier and cohort allocation due to CD34 expression on AML blasts and amount of material available.

Supplemental Table 4B. Comparisons between included and excluded patients.

Supplemental Table 4C. Associations between patients in discovery and validation cohorts.

Supplemental Table 5. Comparisons to assess the impact of batch, instrument, and tissue source on transcript profiles. (separate file due to size)

Supplemental Table 5A: Impact of batch effect on DEGs

Supplemental Table 5B: Impact of sequencing instrument on DEGs

Supplemental Table 5C: Impact of tissue (marrow or blood) on DEGs from MNCs

Supplemental Table 5D: Impact of tissue (marrow or blood) on DEGs from VLBs^CD34+^

Supplemental Table 5E: Overlap of DEGS in MNCs and VLBs^CD34+^

Supplemental Table 5F: DEGs in MNCs vs. VLBs^CD34+^

Supplemental Table 6. Expression changes associated with patient characteristics. (separate file due to size)

Supplemental Table 6A: DEGs associated with gender

Supplemental Table 6B: DEGs associated with age

Supplemental Table 6C: DEGs associated with cytogenetics risk group

Supplemental Table 6D: DEGs associated with ELN risk group

Supplemental Table 7. Pathways associated with DEGs and patient characteristics. (separate file due to size)

Supplemental Table 7A: Pathways associated with DEGs and age in MNCs

Supplemental Table 7B: Pathways associated with DEGs and age in VLBs^CD34+^

Supplemental Table 7C: Pathways associated with DEGs and age in both, MNCs and VLBs^CD34+^

Supplemental Table 7D: Pathways associated with DEGs and cytogenetics in MNCs

Supplemental Table 7E: Pathways associated with DEGs and cytogenetics in VLBs^CD34+^

Supplemental Table 7F: Pathways associated with DEGs and cytogenetics in both, MNCs and VLBs^CD34+^

Supplemental Table 7G: Pathways associated with DEGs and ELN risk in MNCs

Supplemental Table 7H: Pathways associated with DEGs and ELN risk in VLBs^CD34+^

Supplemental Table 7I: Pathways associated with DEGs and ELN risk in both, MNCs and VLBs^CD34+^

Supplemental Table 8. Expression changes and pathways associated with clinical outcomes. (separate file due to size)

Supplemental Table 8A. Pathways associated DEGs and CR, unadjusted.

Supplemental Table 8B. Pathways associated DEGs and CR, adjusted for gender, age and cytogenetics.

Supplemental Table 8C. Pathways associated DEGs and CR, adjusted for ELN risk and age.

Supplemental Table 8D. Pathways associated DEGs and OS, unadjusted.

Supplemental Table 8E. Pathways associated DEGs and OS, adjusted for gender, age and cytogenetics.

Supplemental Table 8F. Pathways associated DEGs and OS, adjusted for ELN risk and age.

Supplemental Table 8G. Validation targets significant in both MNCs and VLBs^CD34+^, adjusted for ELN risk and age.

Supplemental Table 9. Prognostic significance of LSC17 in RNA from MNCs and VLBs^CD34+^ from the discovery cohort.

**Supplemental Methods**

***Identification of Genomic Mutations***

Paired-end reads were first aligned to the human genome reference assembly (GRCh37/hg19) using Burrows-Wheeler Aligner (BWA, v0.7.12) [27]. The resulting alignment data were processed according to the best practice of Genome Analysis Toolkit (GATK, v3.5 https://www.broadinstitute.org/gatk/). Sequence alignment statistics and coverages were computed using Samtools (version 1.0, <http://samtools.sourceforge.net>) and GATK DepthOfCoverage, respectively [28,29]. Variants were called per sample using GATK HaplotypeCaller in GVCF mode, then jointly as a cohort using GenotypeGVCFs. The resulting collection of variants, in the form of a VCF file, were annotated using Annovar (version 2016Feb01) [28,29]. Sequencing depth for each read loci were calculated to determine average percent coverage. For quality control, loci with >20% of samples displaying <65 read coverage were removed from downstream analyses. The following were excluded as potential mutations: a) synonymous variants, b) alterations with low quality (Qual score <150), variant read depth <65 [30], and variant allele frequency (VAF) <10%; and c) alterations in non-exonic loci outside of splice sites. Additional inclusion and exclusion criteria were then applied to the remaining alterations. All nonsense and those missense alterations known to be pathogenic or likely pathogenic in ClinVar were classified as mutations, while missense alterations documented as benign or likely benign were excluded. Missense changes described as having uncertain significance, conflicting interpretation, or not reported in ClinVar were further filtered based on their Provean score, where those changes with a Provean score > -0.25 (i.e., likely nondetrimental) were not included. Three additional missense alterations were deemed not likely pathogenic based on published literature and/or population frequencies [31-33].

***RNA Sequencing for Transcript Biomarkers.***

Paired-end 75 bp reads were sequenced on either Illumina HiSeq 2500 (HiSeq) or NovaSeq 6000 (NovaSeq) instruments (Illumina, San Diego, CA) [35]. For 98% of these libraries, over 40 million read pairs were generated (minimum at 33 million and median at 55 million), which is well above the recommended sequencing depth for detecting differential gene expression [36-39]. RNA-seq data were analyzed by the Fred Hutch Cancer Center Bioinformatics Shared Resource. STAR 2.7.1, with 2-pass mapping, was used to align reads to human reference genome GRCh37/hg19 [40]. FastQC 0.11.8 (<https://www.bioinformatics.babraham.ac.uk/projects/fastqc/>) and RSeQC 3.0.0 were used for QC including insert fragment size, read quality, read duplication rates, gene body coverage and read distribution in different genomic regions [41]. Subread/featureCounts, in Subread 1.6.5, was used to quantify gene-level expression by counting reads in strand-specific mode with respect to GENCODE gene annotation V31lift37 [42]. The expression matrix was processed by edgeR [43]. Genes with zero counts in all samples were first removed and very low expressors were filtered out using function filterByExpr with minimum count of 10, minimum total count of 15. Then the filtered count matrix was normalized using the Trimmed Mean of M-values (TMM) method [43,44]. To reduce the potential false positivity in outcome analyses, a second filtering for low expression was applied to exclude genes with a mean FKPM in the bottom 33% [39]. However, genes within bottom 33% expressors with FPKMs ≥3 in more than 10% of samples were retained, given that the marked molecular heterogeneity of AML leads to some biologically relevant transcripts being expressed at high levels in small subsets of AML specimens [45]. Lists of filtered transcripts and retained, as well as reason for exclusion, are provided in **Supplemental Table 2**. Gene expression between sample groups was compared using GLM LRT method implemented in edgeR, with patient as blocking factor if applicable.

**Quantitative RT/PCR of Transcript Biomarkers**

Taqman gene expression assays were purchased from ThermoFischer Scientific (Waltham, MA, USA). The list of the genes, targeted loci, and assays are provided in **Supplemental Table 3**. Transcript expression was quantified as previous studies [3,6]. The fold change (FC) for each transcript was computed using the comparative Ct method with Beta-glucuronidase (GUSB) normalization to pooled non-malignant bone marrow calibrator for all targets [3,6]. The FC was censored at maximum cycle threshold of 45 for samples without evidence of expression by Q-RT/PCR [45].

**Supplemental Figures**

**Supplemental Figure 1**


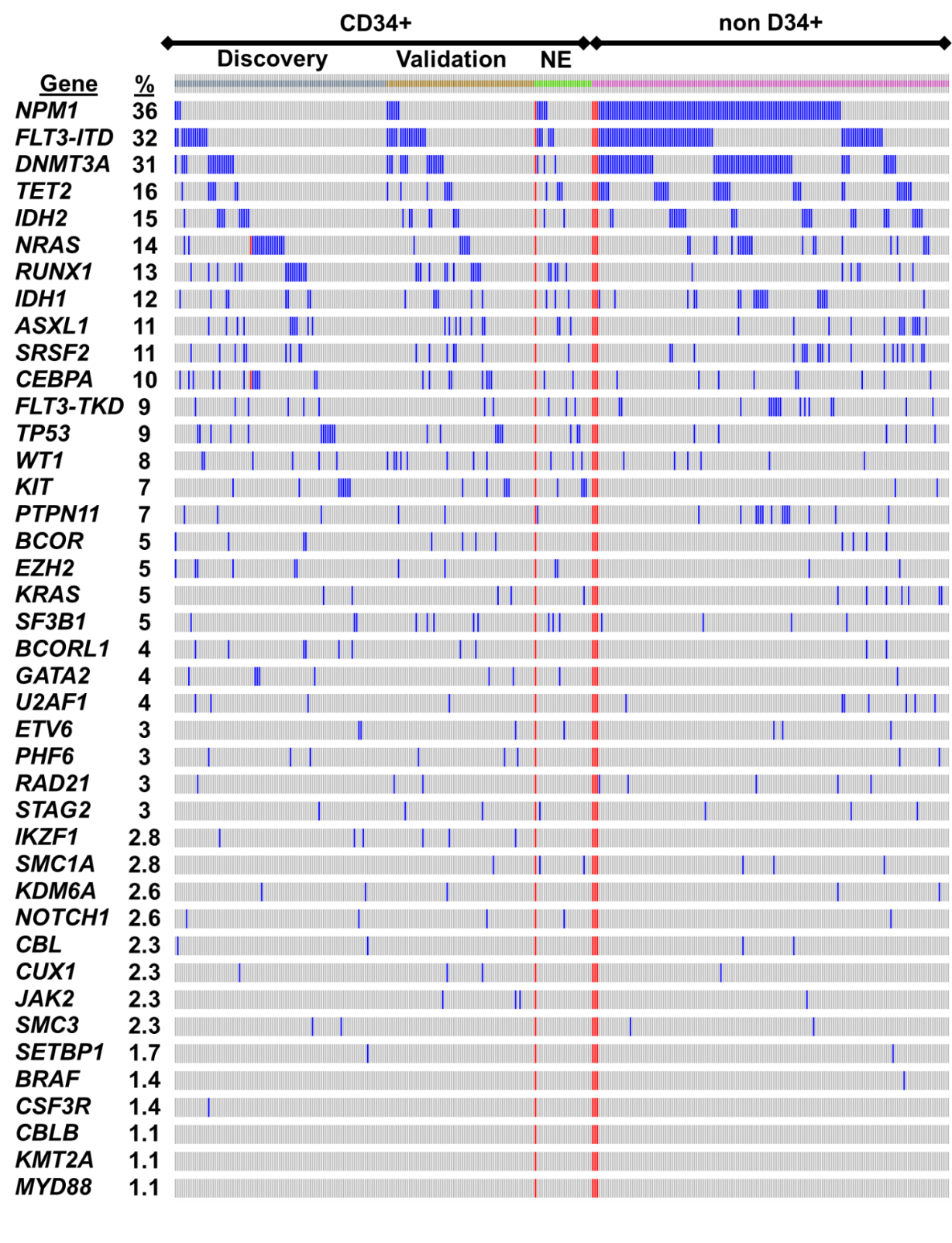


**Supplemental Figure 1**. Figure shows genomic landscape of mutations in various cohorts of patients used for the study. Not evaluated (NE) patients with CD34+ leukemia but inadequate material for RNAseq. Mutation detected (**I**). Mutation data not available (**I**).

**Supplemental Figure 2**

**Supplemental Figure 2. Quality Control Assessments of RNAseq Data.** (A) MD plot displaying log2 fold change in expression profiles between RNA from same specimens (N=4) prepared and sequenced at different time points. (B) MD plot displaying log2 fold change in expression profiles between RNA from same specimens (N=5) prepared and sequenced on different instruments (HiSeq vs. NovaSeq). Decreased expression with HiSeq (blue); Increased expression with HiSeq (red). (C) MD plot displaying log2 fold change in expression profiles between MNC RNA from paired PB and BM specimens (N=3). Decreased expression in BM (blue); Increased expression in BM (red). (D) MD plot displaying log2 fold change in expression profiles between VLB RNA from paired PB and BM specimens (N=3) prepared sequenced at same time. Decreased expression in BM (blue); Increased expression in BM (red).

**Supplemental Figure 3**

**Supplemental Figure 3. Principal Component (PC) Analyses of QC Data**. (A) PC plot displays variance for the normalized RNAseq data from library preps of MNCs from 4 AML patients prepared and sequenced at 3 different time points. (B) PC plot displays variance for the normalized RNAseq data for library preps of MNCs from 5 AML patients sequenced on two different instruments (HiSeq vs. NovaSeq). (C) PC plot displays variance for the normalized RNAseq data from library preps using RNA from MNCs of 3 paired PB and BM AML specimens. (D) PC plot displays variance for the normalized RNAseq data from library preps using RNA from VLB^CD34+^ of 3 paired PB and BM AML specimens.

Patient ID codes: AML25= AML025_P200599775, AML43=AML043_P200771285,

AML48= AML048_P200706491, AML82= AML082_P200986842, AML152= AML152_P199711384, AML166= AML166_P199814282, AML193= AML192_P200506469,

**Supplemental Figure 4**

**Supplemental Figure 4. QQ plots of observed and expected results.** Y-axis represents -log of observed P-values. X-axis represents -log of expected P value. Results for bulk MNCs (MNCs) and VLBs^CD34+^ (VLB) shown in blue and red, respectively. (A) QQ plot for gender. (B) QQ plot for age. (C) QQ plot for cytogenetic risk defined by 4 groups: favorable, intermediate, adverse, and unknown. (D) QQ plot for ELN risk defined by 3 groups: favorable, intermediate, adverse.

**Supplemental Figure 5**

**Supplemental Figure 5. LSC17 score applied to RNAseq data from bulk MNCs and VLBs^CD34+^.** Figures shows overall survival (y-axis) as a function of time (x-axis) stratified by LSC signature using data from the MNCs and VLBs^CD34+^ (VLBs). (A) MNC data without adjusting for ELN and age. (B) VLB^CD34+^ data without adjusting for ELN and age. (C) MNC data with adjusting for ELN and age. (D) VLB^CD34+^ data with adjusting for ELN and age.

**Supplemental Tables**

**Supplemental Tables 1, 2, 4, 5, 6, 7, 8 are provided as separate files.**

**Supplemental Table 3. Taqman assays used for validation.** Chromosomal loci are based on GRCh38 build. All assays were purchased from ThermoFischer Scientific (Waltham, MA).

**Supplemental Table 9.** **Prognostic significance of LSC17 in RNA from MNCs and VLBs^CD34+^ from the discovery cohort.**

*Calculated as the odds ratio per 1-unit increase of the LSC17 score

**Calculated as the hazard ratio per 1-unit increase of the LSC17 score
